# The Role of Sphincterotomy in The Management of Common Bile Duct Stones

**DOI:** 10.1155/1991/47417

**Published:** 1991

**Authors:** H. Neuhaus

**Affiliations:** II. Med. Klinik der TU München Klinikum rechts der Isar Ismaninger Strasse 22, D-800 Miinchen 80, FRG Germany


*HPB Surgery*, 1991, Vol. 3, pp. 211-220
Reprints available directly from the publisher
Photocopying permitted by license only

© 1991 Harwood Academic Publishers GmbH
Printed in the United Kingdom

### HPB INTERNATIONAL

EDITORIAL & ABSTRACTING SERVICE

JOHN TERBLANCHE, EDITOR
Department of Surgery,
Medical School · Observatory 7925
Cape Town · South Africa
Telephone: (021) 47-1250 Ext. 229
Telefax (021) 47-8955

### THE ROLE OF SPHINCTEROTOMY IN THE MANAGEMENT OF COMMON BILE DUCT STONES

**ABSTRACT**

*Neoptolemos JP, Shaw DE, Carr-Locke DL (1989) A Multivariate Analysis of Preoperative Risk Factors in Patients with Common Bile Duct Stones - Implications for Treatment. Annals of Surgery 209: 157–161.*

**A multivariate analysis of 30 preoperative risk factors was undertaken in 248 patients who underwent surgery alone for common bile duct (CBD) stones and in 190 patients who had endoscopic sphincterotomy (ES), 77 of whom subsequently also had surgery. Independently significant risk factors in those undergoing surgery were the serum bilirubin level, the use of preoperatives ES, and the presence of medical risk factors; in patients undergoing ES, only the serum bilirubin and albumin, but not medical risk factors, were of independent significance. The major implications of this study are, first, that high-risk patients should be treated by ES without subsequent surgery, and second, that "fit patients should be treated by surgery alone without routine preoperative ES.**

### PAPER DISCUSSION

KEYWORDS: Biliary tract, gall stones, ERCP

It is currently accepted that endoscopic papillotomy is indicated in all cholecystectomized patients with retained or recurrent bile duct stones. This concept is based on the lower risk of the non surgical management when compared with reoperations [1]. The simple endoscopic technique is effective and shortens the hospitalization period.

In contrast there is a controversial discussion on the indication of endoscopic removal of bile duct stones if the gallbladder if present. In spite of the lack of valid studies that have assessed risk factory for patients with choledocholithiasis, nonsurgical treatment is carried out increasingly. The individual management frequently depends on therapeutic expertise and experience in endoscopic centers. The clear need to identify more objectively and accurately those factors that are associated with posttreatment complications caused NEOPTOLEMOS *et al.* to perform a multivariate analysis of the preoperative risk factors in 438 patients with common bile duct stones [2]. Two hundred and forty eight of these patients underwent surgery alone and in 190 endoscopic papillotomy was carried out, 77 of whom subsequently had surgery as an elective measure or for endoscopic complications. Clinical details of the study were described earlier and are helpful in the interpretation of the results [3]. The multivariate analysis of numerous admission laboratory and clinical variables shows, in particular, that the serum bilirubin level is of independent significance for surgical and endoscopic procedures. This result corresponds to a retrospective analysis of risk factors in patients undergoing surgery for relief of bile duct obstruction [4]. NEOPTOLEMOS additionally assessed the presence of medical risk factors as predictors only of surgical outcome but not of outcome after endoscopic papillotomy (EPT). Further the trial indicates clearly, that preoperative EPT was associated with a higher postoperative complication rate.

The authors conclude from this study that fit patients with common bile duct stones and gallbladder *in situ* should be treated by surgery alone, whereas higher risk patients should be endoscopically treated without subsequent cholecystectomy. The former conclusion is supported by the results of a randomized study of preoperative EPT versus surgery alone for common bile duct stones which showed no benefit for the combined therapeutic strategy [5]. In addition, according to another multivariate analysis of endoscopic and surgical management of bile duct stones the results of EPT were similar to the surgical approach and the risk for the performance of both procedures seems to be additive [6]. These findings are not surprising in view of the high surgical success rates and low mortality rates of less than 1% in fit patients under the age of 60 [1]. Additional risks of routine preoperative EPT would impair the surgical outcome. However, under certain circumstances initial endoscopic common bile duct clearance followed by elective cholecystectomy may be useful. Patients with severe acute biliary pancreatitis or acute cholangitis benefit from this strategy [7,8]. Stones impacted in the distal common bile duct or the so-called ampullary stenosis that might necessitate a transduodenal exploration are probably better managed by preoperative ERC and EPT [9]. In addition in a selected group of eldery patients the preoperative clearance of the bile ducts might lower the overall risks due to the simplification and shortening of the surgical procedure [11]. However, a requirement of this strategy is that the risks of endoscopy are mimimized further. According to the study of NEOPTOLEMOS *et al.* 59% of the post-EPT complications were due to acute cholangitis and septicaemia. The incidence was particularly high in patients in whom initial EPT did not clear the common bile duct. In these cases recently developed techniques like mechanical stone fragmentation as well as intra- or extracorporeal lithotripsy can be safely applied and improve the endoscopic success rates [11]. Even if these procedures are not available or fail, temporary nasobiliary drainage or stenting will establish biliary decompression and permit further elective rather than urgent endoscopic or surgical treatment [12].

The second message of the study is that high risk patients should be considered for endoscopy alone, because this measure is not correlated with admission medical risk factors. Subsequent surgery is hazardous, probably due to the complications and the increased incidence of biliary infections after EPT. Although this risk may be reduced by initial endoscopic stone removal, or at least by drainage procedures, a significant reduction of the mortality rate can only be expected when major surgery and general anesthesia are avoided in this group of high risk patients. Accordingly routine cholecystectomy is not always indicated after successful removal of common bile duct stones.

Various studies showed that the long-term complication rate due to the remaining gallbladder is approximately 10% after EPT which compares favourably with the overall risk for lapatoromy in elderly and frail patients [13]. The evaluation of criteria for the selection of patients who are predisposed to develop gallbladder complications would be of greatest importance. Initial cholangitis or obstruction of the cystic duct were reported as predictors of later gallbladder problems but these findings have unfortunately not been substantiated in larger groups of patients [14,15].

Various techniques of nonsurgical treatment of cholecystolithiasis like ESWL, direct litholysis via percutaneous or transpapillary gallbladder catheterization as well as laparoscopic cholecystectomy or cholecystostomy offer new alternative measures and may prevent long-term complications after endoscopic bile duct clearance. However these procedures have not yet been performed in a large series of high risk patients with previous EPT. Selection criteria and longterm clinical outcome will have to be evaluated and balanced against the endoscopic or surgical strategy based on the results of the LEICESTER-study and comparable unbiased analyses. Finally it should be mentioned that the reported studies have been performed by extremely skillful surgeons and endoscopists. If comparable expertise in the recommended treatment is not available locally, patients may be better managed by an alternative technique or should be referred to a specialist center.

H. Neuhaus
II. Med. Klinik der TU München
Klinikum rechts der Isar
Ismaninger Strasse 22, D-800 München 80, FRG

### REFERENCES

1. Cotton, P.B. (1984) Endoscopic management of bile duct stones; (apples and organges). *Gut*, **25**, 587–597
2. Neoptolemos, J.P., Shaw, D.E. and Carr-Locke, D.L. (1989) A Multivariate Analysis of Preoperative Risk Factors in Patients with Common Bile Duct Stones - Implications for Treatment. *Annals of Surgery*, **209**, 157–161
3. Neoptolemos, J.P., Davidson, B.R. and Shaw, D.E. *et al.* (1987) Study of common bile duct exploration and endoscopic sphincterotomy in a consecutive series of 438 patients., *Br. J. Surg.*, **74**, 916–921
4. Dixon, J.M., Armstrong, C.P., Duffy, S.W. and Davies, G.C. (1983) Factors affecting morbidity and mortality after surgery for obstructive jaundice: a review of 373 patients. *Gut*, **24**, 845–852
5. Neoptolemus, J.P., Carr-Locke, D.L. and Fossard, D.P. (1987) Prospective randomised study of pre-operative endoscopic sphincterotomy versus surgery alone for common bile duct stones. *Br. Med. J.*, **294**, 470–474

6. Miller, B.M., Kozarek, R.A., Ryan, J.A., Ball, T.J. and William Traverso, L. (1987) Surgical Versus Endoscopic Management of Common Bile Duct Stones. *Ann. Surg.*, **207**, 135-141
7. Leese, T., Neoptolemos, J.P., Baker, A.R. and Carr-Locke, D.L. (1986) Management of acute cholangitis and the impact of endoscopic sphincterotomy. *Br. J. Surg.*, **73**, 988–992
8. Neoptolemos, J.P. and Carr-Locke, D.L., London, N.J., Bailey, I.A., James, D. and Fossard D.P. (1988) Controlled trial or urgent endoscopic retrograde cholangio-pancreatography and endoscopic sphincterotomy versus conservative treatment for acute pancreatitis due to gallstones. *Lancet* **II**, 979–983
9. Sheridan, W.G., Williams, H.O.L. and Lewis, M.H. (1987) Morbidity and mortality of common bile duct exploration. *Br. J. Surg.*, **74**, 1095–1099
10. Dresemann, G., Kautz, G. and Bünte, H. (1988) Langzeitergebnisse nach endoskopischer Sphinkterotomie bei Patienten mit Gallenblase *in situ*. *Dtsch. Med. Wschr.*, **113**, 500–505
11. Classen, M., Hagenmüller, F., Knyrim, K. an Frimberger, E. (1988) Giant Bile Duct Stones - Non-Surgical Treatment. *Endoscopy*, **20**, 21-26
12. Cairns, S.R., Dias, L., Cotton, P.B., Salmon, P.R. and Russell, R.C.G. (1989) Additional endoscopic procedures instead of urgent surgery for retained common bile duct stones. *Gut*, **30**, 535–540
13. Dowsett, J.F., Vaira, D., Polydorou, A., Russell, R.O.G. and Salmon, P.R. (1988) Interventional Endoscopy in the Pancreatobiliary Tree. *Am. J. Gastroenterol*, **83**, 1328–1336
14. Davidson, B.R., Neoptolemos, J.P. and Carr-Locke, D.L. (1988) Endoscopic sphincterotomy for common bile duct calculi in patients with gall bladder *in situ* considered unfit for surgery. *Gut*, **29**, 114–120
15. Worthley, C.S. and Toouli, J. (1988) Gallbladder non-filling: an indication for cholecystectomy after endoscopic sphincterotomy. *Br. J. Surg.*, **75**, 796–798

### ENDOSCOPIC TISSUE ADHESIVE FOR GASTRIC VARICES

### ABSTRACT

*Ramond, M-J, Valla, D., Mosnier, J-F, Degott, C., Bernau., J., Rueff, B. and Benhamou, J-P. (1989) Successful Endoscopic Obturation of Gastric Varices with Butyl Cyanoacylate. Hepatology; 10: 488–493.*

**In 27 patients who had bled from esophagogastric varices, large-sized and/or actively bleeding gastric varices were endoscopically obturated with the tissue adhesive butyl cyanoacrylate. Active bleeding was stopped in six patients. Rebleeding occurred in 10 patients; in four patients, rebleeding was due to ruptured gastric varices, occurred early and was successfully treated by reinjection of gastric varices; in one patient, rebleeding was attributed to ulceration on an injected gastric varix. Eight patients died: two of rebleeding (from esophageal varices or undetermined source), four of sepsis and/or liver failure and two at home of undetermined cause. No specific complication due to injection of gastric varices was observed. The results obtained in this series of patients with gastric varices obturated by injection of butyl cyanoacrylate are much more satisfactory than those obtained in previously published series of patients with gastric varices treated by injection of sclerosants.**

